# Effects of Proprioceptive Neuromuscular Facilitation Stretching and Kinesiology Taping on Pelvic Compensation During Double-Knee Extension

**DOI:** 10.1515/hukin-2015-0108

**Published:** 2015-12-30

**Authors:** Seung-Woong Lee, Jung-Hoon Lee

**Affiliations:** 1 Department of Physical Therapy, Inje University, Busan Paik Hospital, Busan, Republic of Korea; 2 Department of Biomedical Health Science, Graduate School, Dong-Eui University, Republic of Korea; 3 Department of Physical Therapy, College of Nursing and Healthcare Sciences, Dong-Eui University, Republic of Korea

**Keywords:** contract-relax with agonist contraction, hamstring shortness, kinesio taping, pelvic compensation, posterior pelvic tilt

## Abstract

Shortened hamstrings are likely to restrict the anterior pelvic tilt and induce a slumped posture due to the posterior pelvic tilt. This study was conducted to compare the effects of proprioceptive neuromuscular facilitation (PNF) stretching and modified anterior pelvic tilt taping (APTT) on hamstring shortness-associated pelvic compensation while executing seated double-knee extension. Male college students (28 healthy young adults; mean age: 21.4 ± 2.1 years) with hamstring shortness were recruited as study subjects and randomly assigned to either the PNF stretching group (control group) or the APTT group (experimental group). In all the subjects, changes in the movement distance of the centre of gluteal pressure (COGP) as well as rectus abdominis (RA) and semitendinosus (SEM) muscle activities were measured during seated double-knee extension while the respective intervention method was applied. Both groups showed significant decreases in COGP distance and RA muscle activity compared with their respective baseline values (p < 0.05), however, no significant changes were observed in SEM muscle activity. We can infer that not only a direct intervention on the hamstring, such as PNF stretching, but also a modified APTT-mediated pelvic intervention may be used as a method for reducing pelvic compensation induced by hamstring shortness.

## Introduction

Prolonged sitting postures owing to excessive length of time spent on the personal computer in the workplace and while studying are major causes of serious imbalance in various muscle groups (Page et al., 2010). Long sitting hours can lead to muscle tissue shortening, especially of the hamstrings due to the compression exerted to the areas below the buttocks and thighs. Thus, shortened hamstrings are likely to restrict the anterior pelvic tilt (Norris, 2001) and induce a slumped posture due to the posterior pelvic tilt, which can ultimately lead to spinal kyphosis (Kuo et al., 2009), leading to extra stress on the tissues around the vertebrae (Norris, 2001). Furthermore, uncontrolled lumbopelvic movement such as a posterior pelvic tilt or lumbar spine flexion may appear before 10–15° of full knee extension can be triggered (Comerford and Mottram, 2012), and compression of hamstrings in a sitting position causes muscle tension due to shortening and myofascial pain in posterior lower legs and the popliteal region (Puranen and Orava, 1988).

Stiffness-induced muscle hypomobility and the consequent reduction of the range of motion (ROM) of the corresponding joints are likely to trigger compensation through the hypermobility of its adjacent segmental muscles (Sahrmann, 2002). With regard to the hamstrings, two joint muscle groups that directly act on hip extension and knee flexion are involved (Kingston, 2005); thus, hamstring stiffness results in reduced hip flexion and knee extension (Neumann, 2013). When executing a double-knee extension to evaluate the degree of reduction in hip extension and knee flexion, the knee joint ROM reduction arising from hamstring stiffness tends to be compensated for by the posterior pelvic tilt (Comerford and Mottram, 2012). Therefore, decreasing the posterior pelvic tilt during seated knee extension while maintaining the neutral zone of the lumbopelvic region (i.e., maintaining an upright lumbar lordosis without any movement of the lumbar vertebrae) may be effective for length testing and selective stretching of the hamstrings (Park et al., 2012). If the lumbopelvic joint is more flexible than the hip joint associated with hamstring extensibility, it may be difficult to maintain the neutral zone of the lumbopelvic region during seated knee extension, which may then lead to a lumbopelvic compensatory motion such as a posterior pelvic tilt and/or lumbar flexion (Sahrmann, 2002).

With regard to increasing hip flexion, stretching of the shortened hamstring muscles may affect lumbar motion during forward bending (Li et al., 1996). Proprioceptive neuromuscular facilitation (PNF) is a conventional manual therapy widely used for improving the flexibility of hamstring muscles (Funk et al., 2003). As a technique for the enhancement of a passive and active joint ROM, PNF is a flexibility training exercise in which active muscle control is achieved through reflexive relaxation of the stretching muscle (Sharman et al., 2006). Decoster et al. (2005) stated that contract-relax with agonist contraction (CRAC) was the most effective PNF technique for increasing the ROM. Comerford and Mottram (2012) introduced a method for controlling compensation (posterior pelvic tilt and/or lumbar flexion) by attaching adhesive sports strapping tape across the uncontrolled segment, thus adopting a very different approach from the conventional interventions that act directly on the hamstring muscles.

Kinesiology taping using elastic adhesive tape, unlike adhesive sports strapping tape, is a new therapeutic method in pain relief, neuromuscular rehabilitation, musculoskeletal disorders, and sports-related injuries. The elastic features of kinesiology tape allow for stretching from a minimum of 120% to a maximum of 170% of its original length, after which it recoils back to its original length (Lee et al., 2014). The mechanism underlying the correction of joint misalignment with kinesiology taping involves the application of elastic adhesive tape with 50–75% of the available tension in the desired position, which provides constant resistance to the misaligned joints and facilitates a rapid return to the desired position (even in conditions of compensation due to incorrect posture) due to the elasticity of the tape (which enables recoil back to its original length) (Lee et al., 2014). A related study demonstrated a mechanical effect of controlling the pelvic alignment deformity caused by habitually assuming a slouching sitting position by using anterior pelvic tilt taping (APTT) that induces the anterior pelvic tilt (Lee and Yoo, 2011).

However, studies that compare between PNF stretching and kinesiology taping, two different approaches to compensation control, are lacking. Against this background, this study aimed to compare changes in pelvic compensation induced by shortened hamstrings while executing double-knee extension, depending on whether PNF stretching or modified APTT is applied.

## Material and Methods

### Subjects

Thirty male college students with hamstring shortness volunteered to participate in this study and signed an informed consent form, which was approved by the institutional review board of the Inje University, before the commencement of the research. The inclusion criteria were as follows: 1) less than 10–15° of full knee extension during active seated knee extension testing for the presence of hamstring shortness, as suggested by Rolls and George (2004), 2) no evidence of knee disease or pain, 3) no neurological or orthopaedic findings in the lumbar and pelvic areas and no history of surgery, 4) no serious deformity or contracture in the joints of the lower extremities, and 5) no contact skin troubles. Two of the 30 volunteers were excluded from the trial as they did not meet the first criterion.

### Procedure

**Figure 1:**
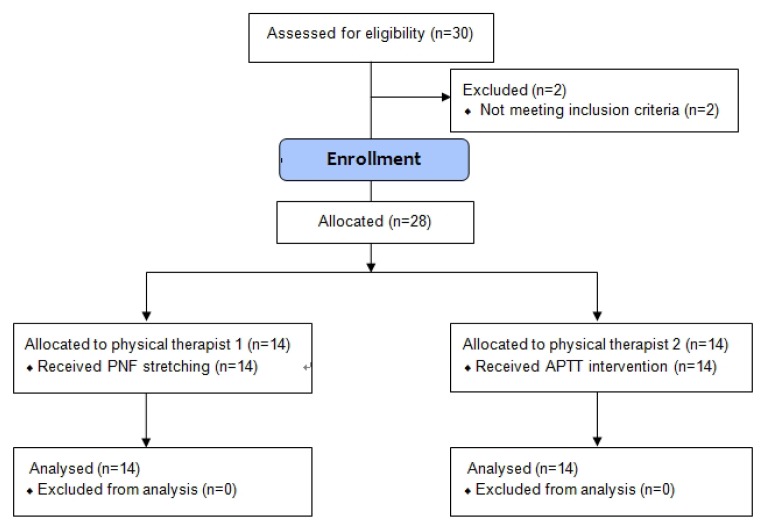
Research design of the study

The study group performed a seated knee extension test. The 28 recruited subjects were randomly assigned to either a PNF stretching group (control group, n = 14) or a modified APTT group (experimental group, n = 14). The study participants in the control and experimental groups were subjected to the PNF_CRAC and modified APTT interventions performed by two physiotherapists (one for each group) specialised in PNF and taping, respectively. Their respective effects were assessed by measuring the changes in the baseline and post-intervention distance of the centre of gluteal pressure (COGP), as well as rectus abdominis (RA) and semitendinosus (SEM) muscle activities during a seated double-knee extension, all of which served as indicators for hamstring shortness (Comerford and Mottram, 2012). Figure 1 presents the flowchart of the procedure.

### Double-knee extension test

**Figure 2:**
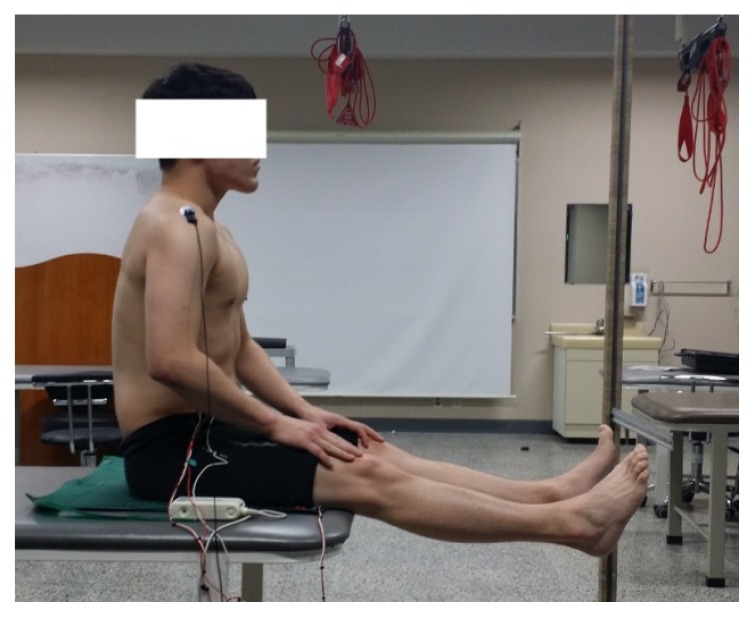
Double-knee extension test

For the double-knee extension test (Figure 2), the uncontrolled movement test (posterior pelvic tilt or lumbar flexion that appears before 10–15° of full knee extension) presented by Comerford and Mottram (2012) was adopted, in which a 90–90° sitting position was assumed, with the knee and hip joints placed so that both feet did not touch the floor; the subject was maintained in a neutral lumbar and pelvic position, with the acromion and ischial tuberosity aligned in a straight line (Comerford and Mottram, 2012). In this posture, both knees were slowly extended and raised up to the end point where no discomfort or pain was felt (Chen et al., 2012), and the height of the foot tip was measured using a rod ruler fixed vertically. During the PNF_CRAC and modified APTT post-intervention measurements, the subjects were instructed to maintain the same foot heights as in the baseline measurements.

### Electromyographic examination

The dominant-side (defined as the preferred leg used to kick a ball (Kramer et al., 1996)) RA and SEM muscle activities during a seated double-knee extension were measured using surface electromyography (sEMG; MP150WSW, BIOPAC System, Inc., CA, USA) with EL503 and Ag/AgCl electrodes. The sampling rate (number of incoming data per second) was fixed at 1000 Hz, with its default stop frequency configured at 60 Hz. Each electromyography signal was recorded twice for 5 s, and the middle 3 s portion was collected (excluding the signal received in the first and last seconds). The root mean square processing was performed for all the collected signals using the programme Acknowledge 3.9.1 (BIOPAC System, Inc.) in units of 250 data.

To obtain accurate data, the muscle areas where electrodes were to be attached to were cleaned with alcohol swabs after rubbing the areas 3 or 4 times with fine sandpaper to remove corneum, followed by disinfection of the haired skin and depilation. We used disposable surface electrodes (Medi-Trace 200; Ludlow Technical Products Canada, Ltd. Canada). One reference electrode was attached to the dominant acromion, and two activity electrodes were attached to the dominant RA and SEM, respectively. Two surface electrodes were attached parallelly to the muscle fibres on the dominant right side as follows: on the RA muscle at approximately 3 cm lateral to the umbilicus, and on the SEM muscle at the centre of a line joining the ischial tuberosity with the medial epicondyle of the tibia (Cram et al., 1998). The maximal voluntary isometric contraction (MVIC) was used as the standard for the action potential (Yoo, 2013). For the measurement of the MVIC of the RA, a straight curl-up was performed from the supine position, and the MVIC of the SEM was measured in a prone position with the knees bent at 90°. MVICs were measured for 5 s in triplicate, and the mean value was determined to be the maximal muscle activity from the 3 s after excluding the first and last seconds.

### COGP distance measurement

For the measurement of the COGP distance during a seated double-knee extension, we used a Tekscan pressure mapping tool (CONFORMat Research 6.20, Tekscan, Inc., South Boston, USA), a sensor mat containing a 44- × 46-cm thin film, with 1,558 tactile sensors attached to it, which can perform static and dynamic measurements of the trajectory changes of the Center of Pressure in a sitting position.

### Intervention

#### Proprioceptive Neuromuscular Facilitation

**Figure 3:**
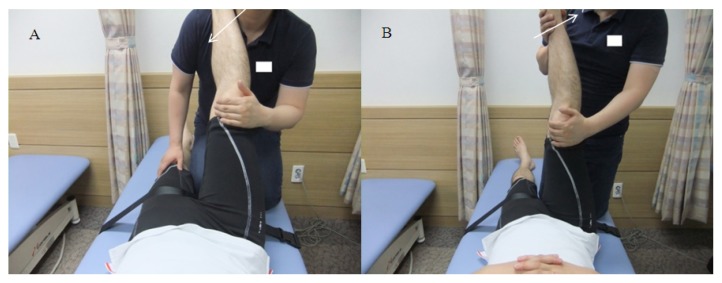
Application of proprioceptive neuromuscular facilitation stretching (contract-relax with agonist contraction)

Among the PNF techniques, the CRAC intervention was applied in this study as it has the greatest capability of inducing significant ROM improvements (Rowlands et al., 2003). Its procedure is described below. In the supine position, with the subject’s uninvolved leg kept immobile with a belt, the physiotherapist slowly raises the involved leg with the knee extended up to the ROM end point (a painless sensation of discomfort) and places it on his shoulder (Chen et al., 2012) (Figure 3A). The subject isometrically contracts his hamstring muscles with almost maximal effort against the resistance offered by the physiotherapist for 10 s, which is followed by a relaxation phase of 10 s; thereafter, the quadriceps muscles are subjected to a concentric contraction again (Chen et al., 2012). During the quadriceps concentric contraction phase, the physiotherapist continues to raise the leg up to the end point of hamstring extension (a painless sensation of discomfort) and keeps it at this state for 10 s (Chen et al., 2012) (Figure 3B). This CRAC cycle was repeated three times on both legs for each subject of the control group.

#### Modified Anterior Pelvic Tilt Taping

All the subjects of the experimental group underwent the modified APTT intervention involving an additional application of the tape from the posterior superior iliac spine to the anterior superior iliac spine along the iliac crest, in the original manner proposed by Lee and Yoo (2011), to induce the anterior pelvic tilt. In addition, to enhance the mechanical correction effect, the second tape was applied on the same areas with approximately 50% overlapping after primary tape application.

**Figure 4:**
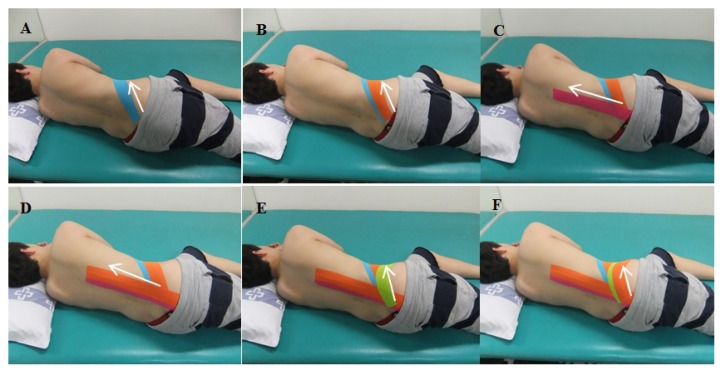
Application of anterior pelvic tilt taping (the posterior part of the body; arrow, direction of tape application)

**Figure 5:**
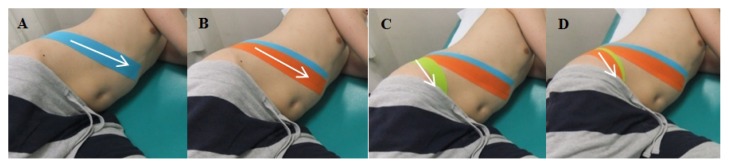
Application of anterior pelvic tilt taping (the anterior part of the body; arrow, direction of tape application)

The main technique used on the target areas was the correctional technique for tilting the pelvis anteriorly. The correctional technique was then applied with 50–60% of tension on the base, middle, and tail of the kinesiology tape (BB Tape, WETAPE Inc., Seoul, Korea) I-Type strip (except for both ends (approximately 2–3 cm) of the tape) while the subject was in a side-lying position, with the involved pelvis tilted anteriorly. While holding the end of the I-Type strip without tension with one hand, 50–60% of tension was applied on the target areas and downward pressure was applied to induce the anterior pelvic tilt. In order to avoid skin irritation, tension was not applied at both ends of the I-Type strip. The I-Type strip was applied twice (with an approximate 50% overlap, as mentioned above) over three different areas, as follows: first, the internal oblique muscle was taped from the posterior superior iliac spine to the xiphoid process underneath the 10–12th ribs (Figure 4A, 4B, 5A, and 5B); second, the erector spinae muscles were taped from the posterior superior iliac spine to the seventh thoracic vertebra along the direction of the muscle fibre (Figures 4C and 4D); the third tape was applied from the posterior superior iliac spine to the anterior superior iliac spine along the iliac crest (Figure 4E, 4F, 5C, and 5D).

### Data analysis

We analysed the values obtained from the PNF_CRAC and modified APTT groups using SPSS 18.0 for Windows (SPSS Inc., Chicago, IL, USA) with the level of significance set at *p* < 0.05. An independent *t-*test was conducted to evaluate the intergroup differences in the changed values regarding the subjects’ general characteristics, COGP distance, and muscle activities. The pre- and post-intervention differences for PNF_CRAC and modified APTT were evaluated with a paired *t-*test.

## Results

In terms of the general characteristics of the subjects of the PNF_CRAC and modified APTT groups, no significant intergroup differences were found (Table 1).

In the PNF_CRAC group, while significant decreases (p < 0.05) were shown in the COGP distance and dominant RA muscle activity, the dominant SEM muscle activity did not show any significant differences (Table 2). The same results were obtained in the modified APTT group regarding COGP distance, and dominant RA and SEM muscle activities (Table 3).

These results indicate that no significant intergroup differences were demonstrated by the PNF_CRAC and modified APTT groups in terms of differences in COGP distance, and dominant RA and SEM muscle activities (Table 4).

## Discussion

This study aimed to compare changes in pelvic compensation induced by shortened hamstrings while executing double-knee extension, depending on whether PNF stretching or modified APTT was applied. The results of this study revealed that the COGP distance decreased significantly in the PNF stretching group. This is considered to have arisen from the effect of PNF stretching that reduced the hamstring shortness-induced posterior pelvic tilt and lumbar flexion by lengthening the sarcomeres of the hamstring muscle, which led to the decrease in COGP distance. Previous studies also reported that CRAC increases length of the hamstring muscles (Chen et al., 2012).

According to the study conducted by Youdas et al. (2010), hamstring muscle activities increased in the relaxation phase of static contraction after the application of the same PNF stretching technique as the one applied in our study. Moreover, Handel et al. (1997) reported that muscle contraction could increase owing to the continuous increase of the number of sarcomeres after the application of PNF stretching. On the contrary, no significant increase in hamstring muscle activities was observed in this study after the PNF stretching intervention. These contradictory results may be ascribed to the different approaches adopted in assessing muscle activities before and after the application of PNF stretching. In the study of Youdas et al. (2010), muscle activities were measured during the state of static isometric contraction after PNF stretching, whereas Handel et al. (1997) measured hamstring muscle activities during the voluntary concentric contraction phase. In this study, we investigated muscle activities during double-knee extension with the hamstring muscles in the lengthened state (i.e., in a state of increased passive tension induced by the lengthened sarcomeres) and no significant changes in muscle activity were noted.

The application of PNF stretching resulted in a significant decrease in dominant RA muscle activities. Such a change implies that the PNF stretching intervention induced a decrease in lumbar flexion because of the reduced posterior pelvic tilt. This means that direct PNF stretching on the shortened hamstring induced a decrease in pelvic compensation, which is likely to occur during a seated double-knee extension, thus, inducing in turn a decrease in COGP distance, which enabled the leg to be raised to the same height despite decreased RA muscle activities.

The modified APTT intervention also resulted in reduced COGP distance and RA muscle activity, however, did not yield any significant differences in dominant SEM muscle activities. Moreover, no intergroup differences were observed in the comparison between the PNF stretching and modified APTT intervention groups. This suggests that the modified APTT-mediated anterior pelvic tilt can induce a decrease in pelvic compensation without any direct intervention on the hamstring muscles, as is the case with PNF stretching. This may be explained by the mechanism of modified APTT, in which kinesiology tape is applied under 50–60% stretching of the original length, thereby yielding resistance against the posterior pelvic tilt while losing 50–60% elasticity already applied when a posterior pelvic tilt occurs to compensate for the shortened hamstring during double-knee extension. Furthermore, the COGP distance might have been also reduced through the maintenance of the decrease in the posterior pelvic tilt enabled by the continuous action of elasticity to regain the original length of the tape driven by increased tension from residual elasticity despite the lengthened tape.

Increased stability of the proximal hamstring and correct alignment can improve the functional movement of the distal hamstring joint (Sahrmann, 2002; Neumann, 2013). Park et al. (2012) reported that stabilising the active neutral position of the lumbar spine and pelvis in a sitting position could minimise the posterior pelvic tilt and influence the change in hamstring length during knee extension. Similarly, in a posture that does not allow a neutral lumbopelvic position, hamstring muscle fibres cannot be effectively lengthened during double-knee extension because of insufficient stabilisation of the proximal hamstring. From this, we can infer that the modified APTT-mediated lumbopelvic stability enabled stabilisation of the proximal hamstring.

As limitations of this study, 5 points may be mentioned. First, the subjects were exclusively male and in their twenties; thus, the study results cannot be applied to other population groups or generalised to the entire population. Second, an intergroup comparison of continuous increasing effects on pelvic compensation could not be performed as the study focused on the immediate effects of the two interventions as demonstrated in seated double-knee extension. Third, only the muscle activities of the dominant side were measured, even though double-knee extension is a bilateral movement. Fourth, the Hawthorne effect, including feedback bias, cannot be controlled as closing the eyes to exclude feedback bias generates postural imbalance, which would affect our gluteal pressure data. Fifth, the activities of muscles (e.g., the erector spinae or abdominal oblique muscles) in the back and pelvis associated with trunk stability could not be measured because the electrode sites were covered with tape.

## Conclusion and Practical Implications

The findings of this study indicate that both interventions, namely PNF stretching and modified APTT using kinesiology tape, reduced the COGP distance and dominant RA muscle activity, and inhibited pelvic compensation. This implies that not only interventions directly applied to the hamstring, such as PNF stretching, but also modified APTT interventions applied to the pelvic region can be used as methods for reducing pelvic compensation induced by hamstring shortness. Therefore, during competitive sports, athletes and coaches can use kinesiology tape as well as PNF stretching in order to reduce pelvic compensation and instability of the lumbopelvic region induced by a shortened hamstring. An increase in hamstring length and lumbopelvic stability may prevent injuries related to hamstring shortness and help in effective movements of the joints that are associated with the hamstring muscle.

## Figures and Tables

**Table 1:** General characteristics of the subjects

Variable	mean±SD		*p*

	PNF_CRAC group (n=14)	Modified APTT group (n=14)	
Age (yrs)	21.9±2.54	20.9±1.69	0.111
Body height (cm)	174.4±4.64	176.8±4.06	0.162
Body mass (kg)	68.1±8.32	69.9±9.66	0.605
SKE of left (°)	55.0±5.88	57.1±8.25	0.437
SKE of right (°)	54.6±5.70	57.1±7.77	0.342

PNF: proprioceptive neuromuscular facilitation; CRAC: contract-relax with agonist contraction; APTT: Anterior pelvic tilt taping; SKE: Seated knee extension

**Table 2:** Comparison between the pre- and post-experiment COGP distance as well RA and SEM muscle activities in the PNF_CRAC group (n = 14)

Variable	mean±SD	

	Pre-PNF_CRAC	Post-PNF_CRAC
COGP distance (cm)	3.7±1.97	2.2±2.22*
RA (MVIC%)	10.5±10.60	6.1±5.20*
SEM (MVIC%)	7.4±7.04	7.5±10.56

COGP: Center of gluteal pressure; RA: Rectus abdominis; SEM: semitendinosus; PNF: proprioceptive neuromuscular facilitation; CRAC: contract-relax with agonist contraction (* p < 0.05)

**Table 3:** Comparison between the pre- and post-experiment COGP distance as well as RA and SEM muscle activities in the modified APTT group (n = 14)

Variable	mean±SD	

	Pre-modified APTT	Post-modified APTT
COGP distance (cm)	4.6±1.99	3.3±1.34*
RA (MVIC%)	5.7±4.19	4.4±2.21*
SEM (MVIC%)	5.9±5.59	8.0±9.44

COGP: Center of gluteal pressure; RA: Rectus abdominis; SEM: semitendinosus; APTT: Anterior pelvic tilt taping (* p < 0.05)

**Table 4:** Comparison between the PNF_CRAC and modified APTT groups in terms of changes in COGP distance as well as RA and SEM muscle activities

Variable	mean±SD		*p*

	PNF_CRAC group	Modified APTT group	
COGP distance (cm)	1.6±2.19	1.3±1.89	0.759
RA (MVIC%)	4.4±5.93	1.3±2.28	0.175
SEM (MVIC%)	−0.1±4.37	−2.2±10.26	0.260

PNF: proprioceptive neuromuscular facilitation; CRAC: contract-relax with agonist contraction; APTT: Anterior pelvic tilt taping; COGP: Center of gluteal pressure; RA: Rectus abdominis; SEM: semitendinosus
